# Assessing Alopecia Areata Management in Poland: Challenges in Medical Practices and Patient Care

**DOI:** 10.3390/jcm15155993

**Published:** 2026-08-01

**Authors:** Julia Hofmann, Łukasz Chętko, Igor Bednarski, Maria Rajczak, Małgorzata Dominiak, Joanna Narbutt, Aleksandra Lesiak

**Affiliations:** 1 Student Scientific Research Club of Experimental, Clinical and Procedural Dermatology, Medical University of Lodz, Pomorska Street 251, 92-213 Lodz, Poland; lukasz.chetko@stud.umed.lodz.pl; 2 Department of Neurology, Medical University of Lodz, ul. Kopcińskiego 22, 90-153 Lodz, Poland; igorbednarskiv@gmail.com; 3 Department of Dermatology, Pediatric Dermatology and Oncology, Medical University of Lodz, Pomorska Street 251, 92-213 Lodz, Poland; maria.rajczak@umed.lodz.pl (M.R.); malgorzata.dominiak@umed.lodz.pl (M.D.); joanna.narbutt@umed.lodz.pl (J.N.); lesiak_ola@interia.pl (A.L.); 4 Dermoklinika Medical Centre S.C. M. Kierstan, J. Narbutt, A. Lesiak, al. Kościuszki, 90-436 Lodz, Poland; 5 Laboratory of Autoinflammatory, Genetic and Rare Skin Disorders, Medical University of Lodz, Pomorska Street 251, 92-213 Lodz, Poland

**Keywords:** alopecia areata, autoimmune diseases, DLQI, JAK inhibitors, ritlecitinib

## Abstract

**Objectives**: Alopecia areata (AA) is a widespread autoimmune condition causing non-scarring hair loss, significantly affecting the quality of life. Despite its prevalence, data on diagnostic and treatment efficacy and the quality of patient care in Poland remain unstudied. The aim of this study was to assess the current clinical approaches to the diagnosis and management of alopecia areata by dermatologists in Poland and to highlight the challenges encountered by Polish patients. **Methods**: A cross-sectional study was conducted regarding dermatologists and AA patients in Poland. The data were gathered from distinct proprietary surveys: an original questionnaire for doctors, and DLQI, CDLQI, AAPPO, WPAI+CIQ:AS, and SF-36 questionnaires for patients. **Results**: The study included 100 dermatologists and 252 patients from Poland. The study revealed that the care provided to patients with alopecia areata is inadequate and lacks a comprehensive approach, despite the negative disease impact on patients’ quality of life. A number of physicians do not follow the diagnostic and treatment guidelines. Despite the registration of next-generation treatments like Janus kinase inhibitors, access to these medications remained limited until recently. **Conclusions**: Alopecia areata in Poland poses significant diagnostic, therapeutic, and psychosocial challenges. While clinical practice largely aligns with international recommendations, notable gaps remain in the use of validated severity tools, psychosocial assessment, and access to advanced therapies. The recent reimbursement of ritlecitinib represents a breakthrough, bringing Polish care in line with global standards. However, optimal management requires not only pharmacological advances but also interdisciplinary strategies integrating psychological support, patient advocacy, and public education to reduce stigma and improve overall quality of life for affected individuals.

## 1. Introduction

Alopecia areata (AA) is a common autoimmune disorder characterized by non-scarring hair loss, affecting approximately 2% of the general population [1,2]. AA exhibits a wide range of clinical manifestations, from small, well-defined areas of hair loss to complete absence of hair on the scalp and body. The condition affects individuals of all ages, genders, and ethnic backgrounds, often leading to frustration due to its unpredictable course, with frequent deteriorations and remissions [3]. Recent discoveries have shed light on the multifactorial etiopathogenesis of AA, encompassing aspects such as genetics, immunology, oxidative stress, the microbiome, allergies, microbiota, epigenetics, and other contributing factors [1,4,5,6,7,8]. Literature reviews indicate that the lifetime risk of developing AA ranges from 0.7% to 3.8% [9]. Although these findings are supported by the literature, there is a lack of epidemiological and demographic data specific to Poland.

According to a 2020 expert consensus, increased risk factors for AA include a family history of the condition and a personal history of other autoimmune disorders such as autoimmune thyroiditis or vitiligo [10,11]. Moreover, numerous studies have reported a notable comorbidity between AA and mental disorders, pointing to the significant roles of stress and psychological factors in the onset and exacerbation of the condition. However, further evidence is required to elucidate the correlations between the immune response, stress, and the physiological factors observed in AA patients [8].

The adverse impact of the disease on patients’ quality of life is well-documented, though data on its effect on various life aspects within the Polish patient population remain scarce. The low public awareness and stigma associated with sudden hair loss may contribute to psychological and psychiatric complications. AA frequently causes considerable psychological distress and negatively affects the quality of life, correlating with increased risks of depression, anxiety, and disturbances in emotional and social functioning. Research on mortality and causes of death in AA patients has shown a significantly higher risk of self-harm and psychiatric conditions compared to control groups [11,12]. Therefore, an early diagnosis, as well as the proper patient care is vital in therapeutic practices.

One of the initial steps emphasized by the team of specialists is the proper assessment of the patient. The diagnosis of alopecia areata can be established on the basis of clinical examination and trichoscopy. The most well-known trichoscopic feature of alopecia areata are exclamation mark hairs which are short hair shafts with a thick and dark distal end. Other trichoscopic markers of alopecia areata activity are black dots, triangular hairs, broken hairs, tapered hairs, and Pohl-Pincus constrictions [11]. One of the most common trichoscopic features of alopecia areata are yellow dots; however, they are not pathognomonic for this condition. The trichogram in the diagnosis of alopecia areata has no diagnostic significance. Scalp biopsy and histological examination are recommended in cases of diagnostic doubt [13,14,15,16,17].

Treatment decisions are guided by the severity of the condition, as measured by the Severity of Alopecia Tool (SALT) or the Alopecia Areata Scale (AAS). For mild cases (SALT < 20), ultra-high potency topical glucocorticoids and intralesional triamcinolone acetonide are the standard treatments, however in many cases they do not provide the required therapeutic effects. In cases of moderate to severe (SALT ≥ 20) alopecia areata, systemic therapy is generally necessary. Currently, the only medications specifically approved for individuals aged 12 and older by the European Medicines Agency (EMA) and the United States are baricitinib and ritlecitinib (JAK 3/TEC inhibitor), which represent a therapeutic breakthrough. Other systemic drugs that may be used off-label for this indication include glucocorticosteroids, cyclosporine, methotrexate, and azathioprine [18,19,20].

Long-term maintenance therapy is of considerable importance in managing alopecia areata. Management demands a multidisciplinary approach and increasing public awareness, which would help improve access to modern and effective treatments. In Poland, the management of alopecia areata has long been hindered by the absence of a dedicated drug program, focusing on off-label treatment [20]. This changed on 1 July 2025, with the introduction of program B.173, which offers free ritlecitinib treatment for patients from 12 years of age with severe disease [21].

Given the scarcity of epidemiological data, the substantial psychosocial burden of alopecia areata, and the recent introduction of a dedicated drug program in Poland, a comprehensive evaluation of current clinical practice is warranted. Importantly, therapeutic strategies have historically relied largely on off-label treatments, and the implementation of targeted therapies represents an early stage of systemic change. Therefore, a real-world assessment of both physician practices and patient experiences is essential to identify existing gaps and support further improvements in the standard of care.

Our study aims to assess the current clinical approaches to the diagnosis and management of alopecia areata by dermatologists in Poland and to highlight the challenges encountered by Polish patients within the context of global standards. The present study was conducted immediately prior to and during the early phase of implementation of the national drug program introducing ritlecitinib. The findings are expected to contribute to better understanding of AA management and enhance the future care of individuals with AA, both in Poland and on a global scale.

## 2. Materials and Methods

### 2.1. Study Design and Participants

A cross-sectional study was conducted in two independent study populations: physicians specializing in dermatology and venereology in Poland and Polish patients with alopecia areata (AA). The physician component assessed diagnostic and therapeutic practices, whereas the patient component assessed disease burden, health-related quality of life, and impairment in work, education, and daily activities. Physicians completed only the original diagnostic and therapeutic practice questionnaire, while patients completed only the patient-reported outcome questionnaires.

Dermatologists working in hospital departments, National Health Fund outpatient clinics, and private practices were invited to complete an original questionnaire concerning diagnostic methods, the use of disease-severity and psychosocial assessment instruments, laboratory investigations, and preferred therapeutic approaches in predefined clinical scenarios. The survey invitation was distributed to dermatologists affiliated with the Polish Dermatological Society via e-mail.

Patients were recruited through the Polish Alopecia Association and the database of the Dermoklinika Medical Centre in Lodz, Poland. Adult and pediatric respondents completed age-appropriate and purpose-specific questionnaires. Eligible patients were informed about the study during follow-up visits or by telephone. Those willing to participate received an anonymous questionnaire by e-mail. All questionnaires were administered anonymously, and participation was voluntary.

Informed consent was obtained from all participants before completion of the questionnaires; in the case of minors, consent was provided by a legal guardian. For both patients and physicians, the first page of the questionnaire contained the participant information sheet and an electronic informed consent statement, with consent provided by selecting the appropriate checkbox before proceeding to the survey.

All study participants were recruited between 15 February and 30 March 2024.

### 2.2. Physician Questionnaire

The physician questionnaire was developed in collaboration with the Regional Consultant in Dermatology and Venereology and critically reviewed by AA management experts affiliated with the Polish Dermatological Society who had been involved in the establishment of the Polish Alopecia Association. It collected demographic and professional information, including sex, years of specialist experience, and workplace. Diagnostic items addressed reliance on medical history and clinical examination, dermoscopy, trichogram, scalp biopsy, mycological testing, laboratory investigations, and the use of validated disease-severity scales. Physicians were also asked whether they used instruments assessing the psychosocial burden of AA and which instruments were selected.

Therapeutic preferences were assessed using clinical scenarios covering limited and severe AA in patients aged <12 years and ≥12 years, as well as involvement of the scalp, eyebrows, eyelashes, and beard. Respondents indicated their first-, second-, and third-line treatment choices. Because the treatment questions contained numerous response categories and several categories had small cell frequencies, therapeutic preferences were analyzed descriptively.

### 2.3. Patient-Reported Outcome Measures

#### 2.3.1. Dermatology Life Quality Index and Children’s Dermatology Life Quality Index

The Dermatology Life Quality Index (DLQI) and Children’s Dermatology Life Quality Index (CDLQI) were used to assess dermatology-specific quality of life in adult and pediatric respondents, respectively. Both instruments contain 10 items scored from 0 to 3, producing total scores from 0 to 30, with higher scores indicating greater impairment. Standard interpretive bands were applied: 0–1, no effect; 2–5, small effect; 6–10, moderate effect; 11–20, very large effect; and 21–30, extremely large effect on quality of life.

In the electronic CDLQI form, the school and holiday alternatives were both visible. In the supplementary scoring analysis, the school response was used when available, and the holiday response was used only when the school response was missing. Sensitivity analyses using alternative handling rules were performed to determine whether this feature materially affected the interpretation of the results.

#### 2.3.2. Alopecia Areata Patient Priority Outcomes

The Alopecia Areata Patient Priority Outcomes (AAPPO) questionnaire was used to assess patient-reported hair loss and the emotional and functional burden of AA. The four hair-loss items—scalp, eyebrows, eyelashes, and body hair—were scored and analyzed separately on ordinal scales ranging from 0 (no hair loss) to 4 (complete hair loss); a combined hair loss score was not calculated. The Emotional Symptoms score was calculated as the mean of Items 5–8, and the Activity Limitations score as the mean of Items 9–11, provided that at least two responses were available within the respective domain. Both domain scores ranged from 0 to 4, with higher scores indicating a greater disease impact.

#### 2.3.3. 36-Item Short Form Health Survey

The 36-Item Short Form Health Survey (SF-36) was used to assess generic health-related quality of life. Responses were recoded so that higher values consistently represented better health or functioning and were linearly transformed to scales ranging from 0 to 100. Eight domains were calculated: Physical Functioning, Role Physical, Bodily Pain, General Health, Vitality, Social Functioning, Role Emotional, and Mental Health. Each domain score was calculated as the mean of its transformed items when at least half of the items within that domain had been answered. The health-transition item was analyzed descriptively and was not included in domain scoring.

The electronic questionnaire used five response categories for the Role Physical and Role Emotional items; these responses were transformed in 25-point increments from 0 to 100. Norm-based Physical Component Summary and Mental Component Summary scores were not calculated because an unequivocal population-specific norm-based scoring algorithm was not available for the questionnaire version used.

#### 2.3.4. Work, Classroom and Activity Impairment

Work productivity, classroom productivity, and regular-activity impairment were assessed using a questionnaire based on the Work Productivity and Activity Impairment framework and adapted to alopecia areata. Work-related outcomes were calculated only for respondents who reported current paid employment, and classroom-related outcomes only for respondents attending school or university.

Work absenteeism was calculated as hours of work missed because of AA divided by usual weekly working hours. Presenteeism was calculated as the self-reported 0–10 work-impairment score divided by 10. Overall work impairment was calculated as absenteeism + [(1 − absenteeism) × presenteeism]. Corresponding formulas were applied to usual class hours, class hours missed, and the 0–10 classroom-impairment score. Regular-activity impairment was calculated as the 0–10 activity–impairment response divided by 10. All outcomes were multiplied by 100 and expressed as percentages, with higher values indicating greater impairment.

### 2.4. Data Collection

Categorical responses were analyzed using the available valid responses for each item. Missing values were not imputed. Patient-reported scale scores were calculated according to the scoring rules specified above, and each analysis used respondents with complete data for the variables involved.

For the adapted productivity questionnaire, short free-text responses containing an unambiguous numeric value were converted to numbers. Written responses indicating no missed time or no impairment were coded as 0, and numeric ranges were represented by their midpoint. Narrative responses that could not be converted reliably, impairment values outside the permitted 0–10 range, and missed-hour values exceeding the corresponding usual hours were treated as missing.

### 2.5. Statistical Analysis

Survey responses were summarized using Microsoft Excel (version 2607, compilation 20228.20110). Statistical analyses were performed using Spotfire Statistica (version 13.3).

Categorical variables were summarized as counts and percentages. Continuous variables were summarized using the mean and standard deviation (SD) and, where informative, the median and interquartile range (IQR). The descriptive values previously reported in the manuscript were retained to preserve consistency with the existing figures, while the additional analyses described below were performed to extend the statistical evaluation.

Associations between specialist experience (<10 years, 10–20 years, and >20 years) and binary clinical-practice outcomes were assessed using Pearson’s chi-square test. When expected cell frequencies were below 5, the Fisher–Freeman–Halton exact test was used. Effect sizes were expressed as Cramér’s V. Because physicians could report more than one workplace, hospital employment, employment in a National Health Fund outpatient clinic, and private-practice employment were coded as separate binary variables. Associations between each workplace and clinical-practice outcomes were assessed using Fisher’s exact test, and odds ratios (ORs) with 95% confidence intervals (CIs) were calculated.

For the clinician analysis, dermoscopy responses of “yes” and “sometimes” were combined as dermoscopy use. Laboratory investigations performed only before planned systemic treatment were not classified as routine laboratory testing in newly diagnosed patients. Treatment preferences were summarized descriptively because the large number of treatment categories and small cell frequencies precluded stable inferential comparisons.

Internal consistency of the AAPPO Emotional Symptoms and Activity Limitations domains, DLQI, CDLQI, and the eight SF-36 domains was assessed using Cronbach’s alpha. Cronbach’s alpha was not calculated for the productivity outcomes because absenteeism, presenteeism, overall impairment, and activity impairment represent distinct constructs rather than interchangeable items.

Associations between ordinal or non-normally distributed questionnaire outcomes were evaluated using Spearman’s rank correlation coefficient. Differences in AAPPO Emotional Symptoms and Activity Limitations scores across the five response categories of each hair-loss item were explored using the Kruskal–Wallis test, with epsilon-squared reported as the effect-size estimate.

For cross-questionnaire analyses, anonymous patient questionnaires were linked chronologically because Microsoft Forms response numbers restarted independently in each form and therefore did not constitute a shared participant identifier. AAPPO was used as the anchor dataset. DLQI, CDLQI, SF-36, and productivity-questionnaire responses were matched one-to-one while preserving chronological order, and a match was accepted only when questionnaire start times differed by no more than 30 minutes. Each response could be used only once. Cross-questionnaire correlations required at least 10 complete linked pairs and variability in both variables.

The Benjamini–Hochberg procedure was applied within prespecified families of related analyses to control the false discovery rate. All statistical tests were two-sided. An adjusted *p* value < 0.05 was considered statistically significant. The analyses were exploratory and cross-sectional; therefore, no causal inference was made.

## 3. Results

The physician survey included 100 dermatology and venereology specialists. Patient questionnaire sample sizes differed by instrument because the questionnaires were administered separately and available-case analyses were used: 105 respondents completed the DLQI, 89 completed the CDLQI, 277 completed the AAPPO, 187 completed the SF-36, and 171 completed the WPAI-derived questionnaire.

### 3.1. Dermatological Practices and Treatment Patterns

**Figure 1:**
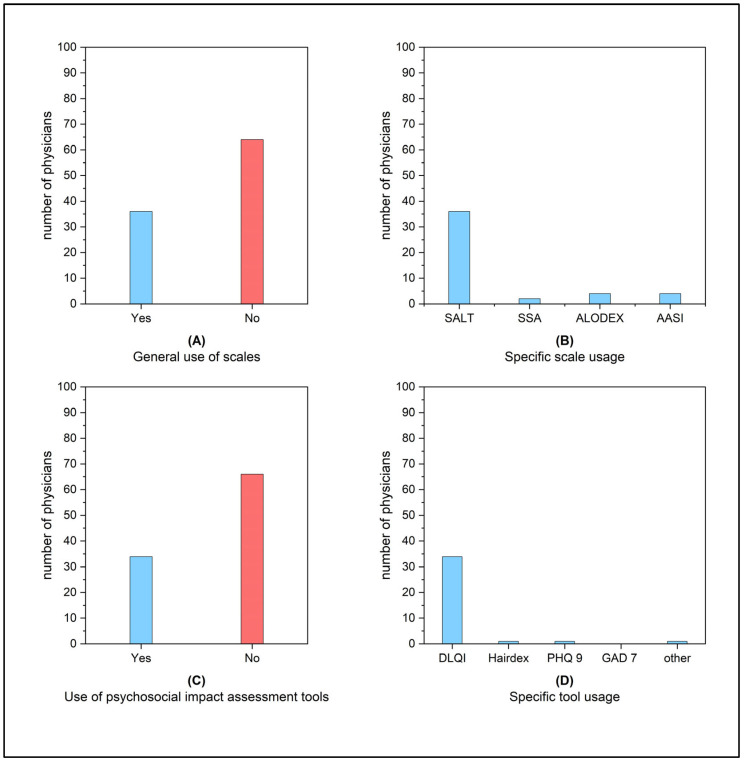
Use of disease activity scales and psychosocial assessment tools among the surveyed physicians: (**A**) general use of scales; (**B**) specific disease activity scale usage; (**C**) general use of psychosocial impact assessment tools; (**D**) specific psychosocial impact assessment tool usage.

The physician sample comprised 80 women and 20 men working in different medical settings; 33% had more than 20 years of specialist experience. Diagnostic practices varied. Thirty-nine percent diagnosed alopecia areata using medical history and clinical examination alone, whereas 78% used dermoscopy. Biopsy and trichogram were reported by 5% and 13% of physicians, respectively. Disease-severity scales were used by 36% of respondents, most commonly the Severity of Alopecia Tool (SALT), and 35% used instruments assessing psychosocial burden, most frequently the DLQI (Figure 1A–D).

**Figure 2:**
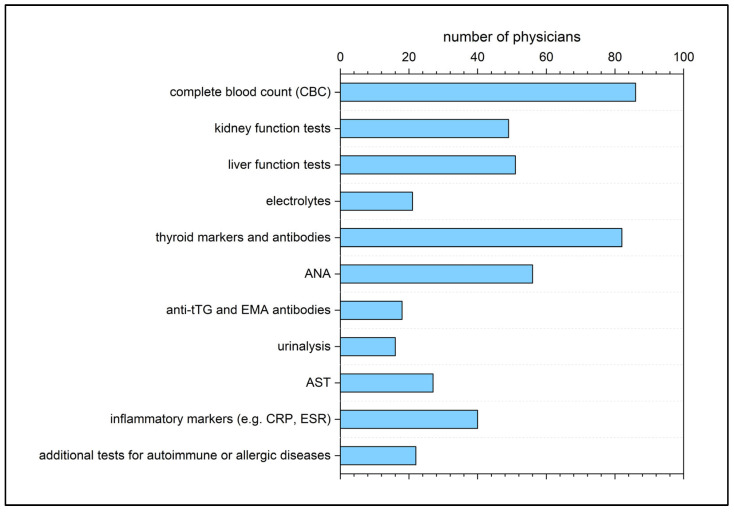
Routine laboratory tests ordered by physicians.

The most frequently ordered diagnostic tests by physicians included complete blood count, thyroid function tests and autoantibodies, antinuclear antibodies, as well as assessments of liver and kidney function (Figure 2).

Years of specialist experience were significantly associated with diagnosis based solely on clinical examination and history (χ^2^ = 16.11, *p* < 0.001, Cramér’s V = 0.41), dermoscopy use (χ^2^ = 19.98, *p* < 0.001, V = 0.46), use of disease-severity scales (χ^2^ = 19.44, *p* < 0.001, V = 0.45), use of psychosocial assessment tools (χ^2^ = 19.44, *p* < 0.001, V = 0.45), and use of the SALT scale (χ^2^ = 19.01, *p* < 0.001, V = 0.44). Dermoscopy, severity scales, psychosocial tools, and SALT were used most frequently by physicians with less than 10 years of specialist experience. No significant experience-related differences were found for biopsy, trichogram, routine mycological testing, or routine laboratory testing.

Workplace was also associated with several clinical practices. Hospital-based physicians had higher odds of using psychosocial assessment tools (OR = 11.00), disease-severity scales (OR = 10.12), and dermoscopy (OR = 8.80); all associations remained significant after false-discovery-rate correction. Physicians working in National Health Fund outpatient clinics had lower odds of using the SALT scale (OR = 0.23, 95% CI 0.09–0.55, adjusted *p* = 0.005), disease-severity scales (OR = 0.24, 95% CI 0.10–0.58, adjusted *p* = 0.007), and dermoscopy (OR = 0.12, 95% CI 0.03–0.55, adjusted *p* = 0.008). Private-practice work was positively associated with dermoscopy use (OR = 8.36).

In limited alopecia areata, therapeutic choices were similar across age groups. Topical glucocorticosteroids were the predominant first-line treatment in patients aged ≥12 years (61.22%, *n* = 60) and in children aged <12 years (62.89%, *n* = 61). A contact sensitizer or irritant combined with a topical corticosteroid was the second most frequent choice (16.33%, *n* = 16 and 13.40%, *n* = 13, respectively).

For beard involvement, topical corticosteroids were the most frequently selected treatment (57.14%, *n* = 56), followed by topical calcineurin inhibitors (17.35%, *n* = 17) and a combination of topical corticosteroids and calcineurin inhibitors (12.24%, *n* = 12). For eyebrow involvement, topical corticosteroids remained the leading option in patients aged ≥12 years (51.02%, *n* = 50) and <12 years (45.45%, *n* = 45), although calcineurin inhibitors were reported relatively frequently in both groups (25.51%, *n* = 25 and 35.35%, *n* = 35, respectively).

In severe alopecia areata, topical corticosteroids remained the most frequent choice in children aged <12 years (34.38%, *n* = 33), whereas systemic glucocorticosteroids were the leading choice in patients aged ≥12 years (29.47%, *n* = 28), followed by topical corticosteroids (18.85%, *n* = 18) and cyclosporine (13.68%, *n* = 13). In severe generalized alopecia areata, systemic glucocorticosteroids were the most frequently reported option in both children (33.68%, *n* = 32) and patients aged ≥12 years (45.16%, *n* = 42), followed by cyclosporine (16.84%, *n* = 16 and 19.35%, *n* = 18, respectively).

**Figure A1:**
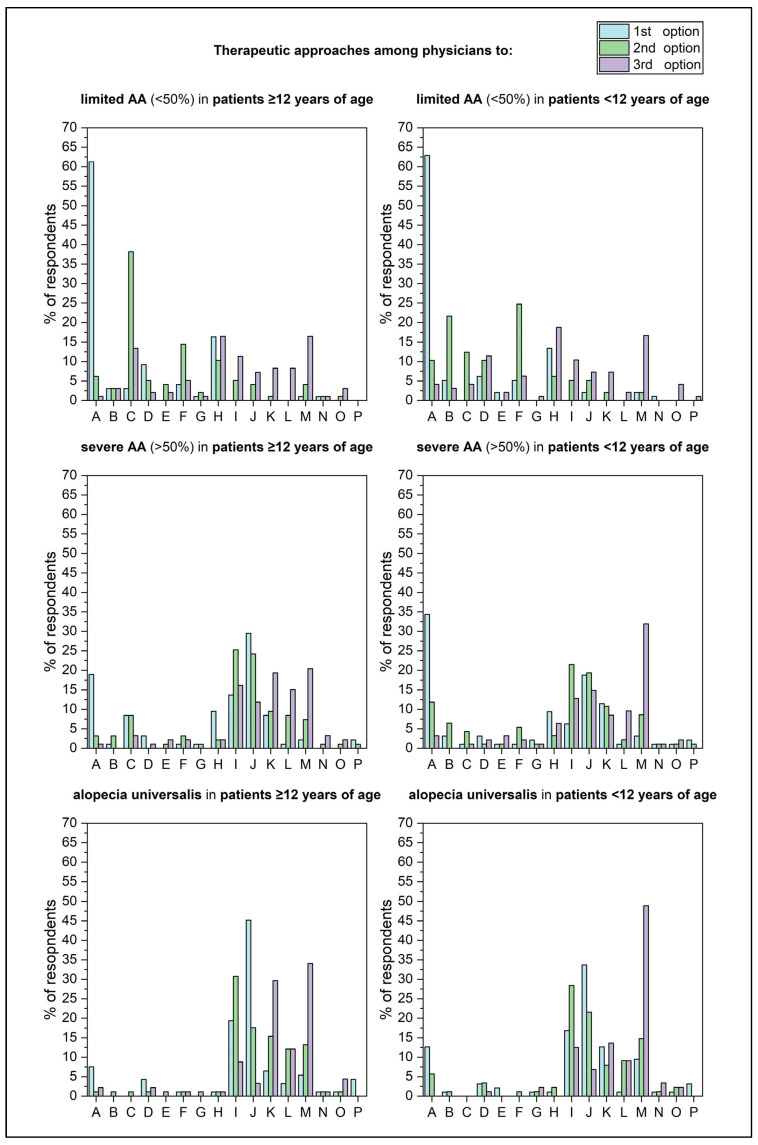
Therapeutic approaches among physicians to specific AA cases: (A), topical corticosteroids; (B), topical calcineurin inhibitors; (C), intralesional steroid injections; (D), combination therapy: a topical corticosteroid and a topical calcineurin inhibitor; (E), contact sensitizers (ex. DPCP, SADBE); (F), contact irritants (ex. anthralin, dithranol); (G), combination therapy: a contact sensitizer and an irritant; (H), combination therapy: a contact sensitizer or an irritant and a topical corticosteroid; (I), systemic cyclosporine; (J), systemic corticosteroids; (K), combination therapy: systemic cyclosporine and systemic corticosteroids; (L), systemic JAK inhibitors; (M), “I don’t use any treatment”; (N), other; (O), systemic methotrexate; (P), phototherapy.

Use of Janus kinase inhibitors was rare in the historical treatment data: 1.05% (*n* = 1) in severe disease among patients aged ≥12 years, 1.04% (*n* = 1) in children aged <12 years, and 3.23% (*n* = 3) in severe generalized disease among patients aged ≥12 years. Complete therapeutic distributions are presented in Figure A1, and their concordance with clinical recommendations is summarized in Table A1.

### 3.2. Dermatology-Specific Quality of Life: DLQI and CDLQI

**Figure 3:**
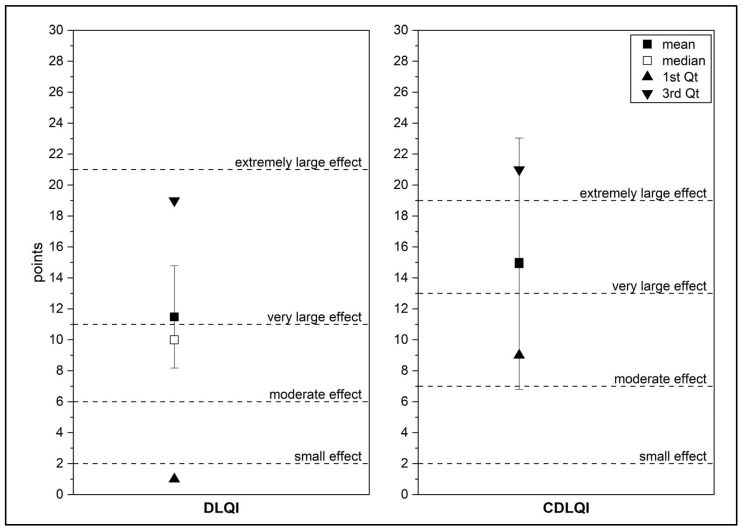
Mean and median DLQI and CDLQI scores among patients with AA.

**Figure 4:**
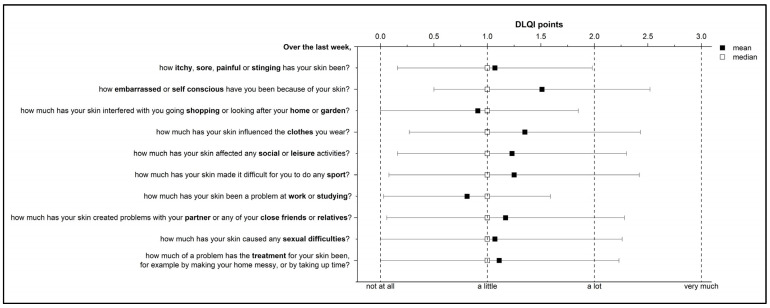
Item-by-item analysis of the DLQI scores among patients with AA.

The original descriptive analysis demonstrated substantial dermatology-specific quality-of-life impairment. The mean DLQI score reported in the original analysis was 12.33. Thirty-two percent of adult respondents reported a very severe effect, 21% a severe effect, and 15% a moderate effect. Embarrassment, interference with clothing and social activities, limitations in sport, and difficulties with everyday tasks were among the most frequently reported problems (Figure 3 and Figure 4).

**Figure 5:**
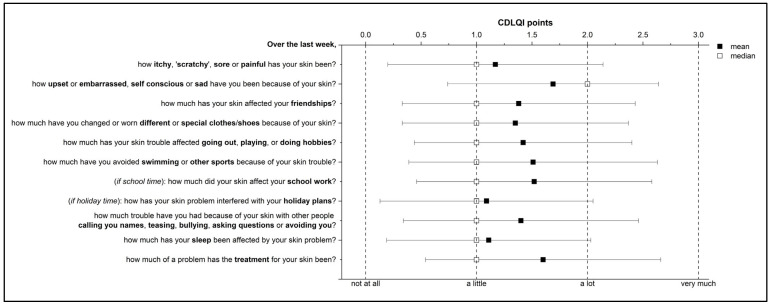
Item-by-item analysis of the CDLQI scores among patients with AA.

The mean CDLQI score reported in the original analysis was 14.48. Among pediatric respondents, 87% reported shame, shyness, sadness, or anxiety related to alopecia areata, 44% reported difficulties with friends, 42% reported teasing, bullying, or social avoidance, and 24% reported school-attendance problems during the preceding week (Figure 3 and Figure 5).

In the supplementary psychometric analysis, internal consistency was excellent for both instruments (Cronbach’s α = 0.94 for the DLQI and α = 0.92 for the CDLQI). The highest-scoring DLQI item concerned embarrassment, whereas the highest-scoring CDLQI item concerned embarrassment, sadness, or worry.

Sensitivity analyses addressing simultaneous completion of the school and holiday alternatives in the electronic CDLQI form did not materially change the interpretation. Using the higher of the two responses yielded a mean total score of 14.22, whereas treating the dual response as one missing item yielded a mean of 13.18; both approaches confirmed substantial pediatric quality-of-life impairment.

### 3.3. Alopecia Areata Patient Priority Outcomes

**Figure 6:**
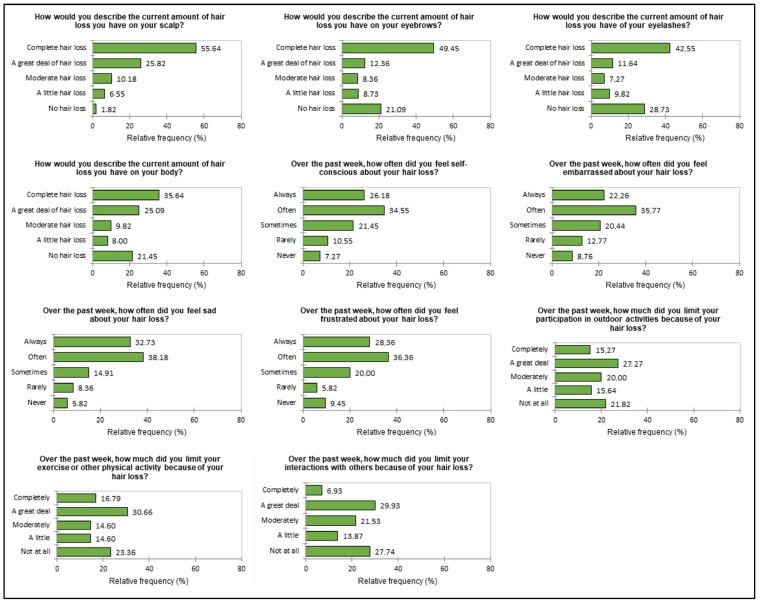
Item-by-item analysis of the AAPPO questionnaire results.

The item-level AAPPO results showed extensive hair loss. Complete hair loss was reported for the scalp by 55.64% of respondents, for the eyebrows by 49.45%, for the eyelashes by 42.55%, and for body hair by 35.64%. Severe hair loss was reported by 25.82% for the scalp, 12.36% for the eyebrows, 11.64% for the eyelashes, and 25.09% for body hair. The complete item-level distribution is presented in Figure 6.

Psychological distress was frequent. Overall, 34.55% of respondents often and 26.18% always felt self-conscious; 38.18% often and 32.73% always felt sad; 36.36% often and 28.36% always felt frustrated; and 35.77% often and 22.26% always felt embarrassed because of hair loss. Outdoor activities, exercise, and social interactions were also frequently limited.

Supplementary domain scoring yielded a mean AAPPO Emotional Symptoms score of 2.66 ± 1.09 (median 3.00, IQR 2.00–3.50) and a mean Activity Limitations score of 1.93 ± 1.29 (median 2.33, IQR 0.67–3.00). Internal consistency was excellent for Emotional Symptoms (Cronbach’s α = 0.94) and Activity Limitations (α = 0.92). The two domains were strongly correlated (Spearman’s ρ = 0.742, adjusted *p* < 0.001).

Associations between the four anatomical hair-loss items and the Emotional Symptoms or Activity Limitations domains were very weak, and none remained statistically significant after false-discovery-rate correction. Exploratory differences across scalp hair-loss categories had only small effect sizes (ε^2^ approximately 0.05–0.06) and were not consistently reproduced for eyebrow, eyelash, or body hair loss.

### 3.4. Generic Health-Related Quality of Life: SF-36

The original item-level analysis showed that 40.11% of respondents rated their overall health as good. Physical or psychological health negatively affected social functioning in 47%, while nervousness, irritability, depressed mood, and fatigue were frequently reported. Overall, 57% stated that alopecia-related hair loss negatively affected mood and physical activity, and 35% reported substantial or complete restriction of social interactions.

In the domain-level analysis, SF-36 data were available for 187 respondents, and all eight domain scores were calculable for at least 186 participants. Physical Functioning had the highest mean score (79.95 ± 24.09), followed by Bodily Pain (64.57 ± 27.12) and Role Physical (58.24 ± 28.75). Lower mean scores were observed for Role Emotional (48.34 ± 30.91), Social Functioning (46.19 ± 27.25), Mental Health (44.10 ± 20.36), General Health (42.14 ± 20.52), and Vitality (39.91 ± 21.80).

This profile indicated relatively preserved basic physical functioning but greater impairment in energy, perceived general health, mental well-being, social functioning, and role functioning. Internal consistency was acceptable to excellent across the eight SF-36 domains, ranging from α = 0.72 for General Health to α = 0.96 for Role Emotional.

Item-by-item SF-36 questionnaire results can be found in Supplement S1 in the Supplementary Material.

### 3.5. Work, Classroom and Activity Impairment: WPAI+CIQ

The original descriptive analysis showed a substantial occupational and educational burden. Among economically active respondents, 34% reported disease-related work interruption during the preceding week, with an average of 11.75 work hours lost and a maximum of 40 h. Among 76 student respondents, 38% reported school or class absence, with an average of 8 h lost and a maximum of 30 h.

Among 171 respondents to the adapted productivity questionnaire, 98 reported current paid employment and 71 reported attending school or university; some respondents belonged to both groups. Standardized work absenteeism averaged 9.25 ± 20.52%, work presenteeism 34.85 ± 34.25%, and overall work impairment 36.08 ± 34.87%. Class absenteeism averaged 14.41 ± 23.66%, impairment while attending classes 44.20 ± 33.76%, and overall classroom impairment 46.91 ± 36.97%. Regular-activity impairment averaged 40.91 ± 32.83%.

Formal absence was frequently zero, whereas impairment while present at work or school was substantial. Consequently, absenteeism alone underestimated the functional burden of alopecia areata.

### 3.6. Associations Between Patient-Reported Outcomes

Chronological one-to-one linkage matched more than 94% of each patient-questionnaire dataset to an AAPPO response within the prespecified 30-minute window. The linked analyses demonstrated convergence across disease-specific, dermatology-specific, generic health-status, and productivity measures.

AAPPO Activity Limitations showed the strongest association with SF-36 Social Functioning (ρ = −0.616, *n* = 185, adjusted *p* < 0.001). AAPPO Emotional Symptoms were also inversely associated with Social Functioning (ρ = −0.556, *n* = 185, adjusted *p* < 0.001). Activity Limitations were associated with poorer Mental Health (ρ = −0.461), Role Emotional (ρ = −0.446), Role Physical (ρ = −0.390), and Vitality (ρ = −0.380); all associations remained significant after false-discovery-rate correction.

Greater AAPPO Activity Limitations were associated with greater regular-activity impairment (ρ = 0.417, *n* = 157, adjusted *p* < 0.001). In adults, the DLQI was strongly associated with work presenteeism (ρ = 0.639, *n* = 63, adjusted *p* < 0.001) and overall work impairment (ρ = 0.634, *n* = 60, adjusted *p* < 0.001). Poorer SF-36 Role Physical and Role Emotional scores were associated with greater regular-activity impairment (ρ = −0.477 and −0.464, respectively; both adjusted *p* < 0.001).

Associations involving absenteeism were generally weaker and less consistent than those involving presenteeism, overall productivity impairment, or regular-activity impairment. Overall, greater emotional and activity-related burden was most consistently associated with poorer mental and social functioning and with reduced effectiveness during work, education, and regular daily activities.

## 4. Discussion

Alopecia areata (AA) is recognized as a significant dermatological condition worldwide, with substantial medical, psychological, and socioeconomic consequences [22]. The most comprehensive epidemiological analysis to date, covering the period from 1990 to 2021, demonstrated that while the absolute number of AA cases has risen globally, the age-standardized incidence rate (ASIR) has remained largely stable or has slightly declined [1,2,3,4,22]. Higher ASIRs are consistently observed in high-income countries, likely reflecting improved recognition and more systematic data collection, whereas increasing incidence in developing regions is attributed to better healthcare access and diagnostic practices. Despite these advances, country-specific data from Poland remain limited, highlighting the value of our study in providing contemporary insights into AA management. Beyond providing an overview of current clinical practice, the present study offers novel insights into factors associated with diagnostic behaviour and patient-reported disease burden in alopecia areata.

Diagnostic strategies in AA have been extensively discussed in international consensus statements, with most guidelines emphasizing the central role of clinical examination supplemented by dermoscopy [11,14,17]. Trichoscopy is now considered the standard of care in differentiating AA from other non-scarring alopecias, and validated severity assessment tools such as the SALT or the AAS are recommended for both clinical and research purposes [1,4,23]. Our findings confirm strong adherence to trichoscopy-based diagnosis in Poland, with 78% of physicians employing dermoscopy at the initial stage, which aligns well with global best practices. However, 13% of physicians still reported using trichograms, despite their limited diagnostic value, which points to the persistence of outdated methodologies. Even more concerning is the underutilization of validated severity tools, as only a minority of respondents applied scales such as SALT or AAS in routine practice. This underuse can be attributed to several practical barriers. First, the limited duration of outpatient dermatology consultations in the public sector resulting from the high patient volume and limited availability of dermatologists may at times leave little room for standardized severity scoring and psychosocial burden assessment after clinical examination, treatment discussion, and documentation have been completed. Qualitative studies have confirmed that dermatologists identify time constraints as a primary barrier to integrating standardized outcome measures into daily practice, and even small additions to encounter time can produce disproportionate downstream effects—a simulation study demonstrated that adding just 2 min of documentation per visit increased average patient wait times by 41% [24]. Secondly, until the recent introduction of the Polish National Health Fund (NFZ) drug program for AA—which mandates SALT score documentation as a criterion for eligibility for reimbursed JAK inhibitor therapy—there was no systemic incentive for routine severity scoring in clinical practice. The absence of prior reimbursement-linked requirements likely contributed to the underuse of validated severity instruments, as clinicians had little practical motivation to incorporate these time-consuming assessments into their routine clinical practice. Of note, inferential analyses further demonstrated that physician experience and workplace significantly influenced adherence to guideline-recommended diagnostic practices. Dermatologists with shorter specialist experience more frequently used dermoscopy, validated severity scales, and psychosocial assessment tools, possibly reflecting greater familiarity with recently published recommendations and structured assessment methods. Likewise, hospital-based physicians were more likely to perform standardized disease assessment than those working predominantly in outpatient settings. These findings suggest that implementation of evidence-based diagnostic strategies is influenced by both educational background and clinical environment, highlighting potential targets for future educational initiatives.

Therapeutic approaches to AA remain heterogeneous worldwide. International recommendations advocate topical corticosteroids as first-line treatment for limited disease, whereas systemic therapies are reserved for more severe cases [11,18,20]. Our results indicate that therapeutic strategies used in Poland are generally consistent with these recommendations, despite the absence of dedicated national guidelines. Topical corticosteroids remained the preferred first-line treatment for limited AA, whereas systemic glucocorticosteroids and cyclosporine predominated in severe disease.

Inferential statistical analyses of therapeutic preferences were intentionally not performed because physicians could select multiple treatment options across numerous clinical scenarios, resulting in sparse contingency tables and unstable estimates. Accordingly, treatment patterns were analysed descriptively to provide a reliable overview of current clinical practice without overinterpreting statistically underpowered comparisons.

Nevertheless, several deviations from international recommendations were observed. Calcineurin inhibitors were prescribed relatively frequently, particularly for eyebrow involvement, despite limited evidence supporting their efficacy. Contact immunotherapy was commonly introduced earlier than recommended, and intralesional corticosteroids were occasionally used in children younger than 12 years. Overall, these findings indicate that implementation of evidence-based recommendations remains heterogeneous despite the availability of international consensus documents, emphasizing the need for standardized clinical algorithms and continued medical education.

The most pronounced therapeutic gap identified in our study concerns the limited use of Janus kinase (JAK) inhibitors. Although JAK inhibitors have become the preferred targeted therapy for severe AA following robust clinical evidence and regulatory approval [25], access to these agents in Poland remained extremely limited because reimbursement was unavailable during the study period. Consequently, only four physicians reported prescribing JAK inhibitors.

The low frequency of JAK inhibitor use should therefore be interpreted primarily as a consequence of historical reimbursement restrictions rather than limited physician acceptance of targeted therapy. Because the study was conducted immediately before and during the introduction of the national reimbursement programme, it provides an important pre-implementation benchmark for future evaluations of prescribing patterns, access to targeted therapies, and patient outcomes following reimbursement.

Beyond pharmacological treatment, AA exerts a profound psychosocial burden that is frequently underestimated in routine clinical practice. Although international studies consistently identify AA as one of the dermatological diseases with the greatest impact on quality of life, only 35% of physicians in our study routinely assessed psychosocial burden using validated questionnaires. Children demonstrated particularly severe impairment, whereas adults experienced substantial occupational and emotional consequences. These findings are consistent with previous reports describing AA as a chronic disease with significant psychosocial, educational, and socioeconomic impact.

Correlation analyses further demonstrated consistent relationships between dermatology-specific quality of life, emotional burden, general health, and work productivity. Greater impairment in DLQI was accompanied by poorer overall functioning, whereas higher emotional burden measured by AAPPO was associated with greater activity limitations and occupational impairment. Together, these findings emphasize the multidimensional nature of disease burden in alopecia areata.

The observed correlations further indicate that the applied patient-reported outcome measures provide complementary rather than redundant information. While the DLQI evaluates dermatology-specific quality of life, the AAPPO captures disease-specific emotional burden, and the SF-36 and WPAI assess general health and functional impairment. Routine use of multiple validated questionnaires may therefore provide a more comprehensive patient-centred assessment than reliance on a single instrument. The excellent internal consistency demonstrated by all patient-reported outcome measures confirms the reliability of the applied questionnaires within the Polish AA population. These findings support their use in both future observational studies and routine clinical practice, where standardized patient-reported outcomes may facilitate longitudinal monitoring and more patient-centred therapeutic decision-making.

Stigmatization of individuals with AA remains an important public health challenge. Public misconceptions frequently lead to social isolation and trivialization of the disease as merely a cosmetic condition. International initiatives, including those led by the National Alopecia Areata Foundation (NAAF) and the American Academy of Dermatology (AAD), illustrate how education and patient advocacy may reduce stigma [26]. Comparable initiatives remain limited in Poland, highlighting the need for broader public education and stronger multidisciplinary support.

Taken together, our findings underscore that improving outcomes in AA requires a holistic and integrated model of care that combines evidence-based treatment, standardized care, psychosocial support, and public health strategies aimed at reducing stigma and improving disease awareness. To address the gaps highlighted by our research, several targeted initiatives could be considered. Firstly, extending specialist consultation time is essential in order to allow the routine use of validated severity assessment tools and comprehensive patient counseling. Secondly, general practitioners (GPs) play a critical role as the first point of contact for patients with hair loss, particularly in rural and underserved areas where access to dermatologists is limited. Targeted educational initiatives for GPs—who serve as health promoters, educators, and gatekeepers for specialist referral—could facilitate earlier recognition of AA and more timely referral, enabling earlier treatment initiation and potentially improving patient outcomes. Moreover, collaboration between patient advocacy organizations and professional dermatological societies should be strengthened. The model established by the NAAF, which organizes biennial research summits that bring together scientists, clinicians, and patient advocates, demonstrates how such partnerships can accelerate both research and clinical education [27]. In Poland, where physicians are obliged to participate in continuing professional development, incorporating such AA-focused sessions into national dermatological congresses and conferences would ensure that practicing clinicians remain updated on evolving diagnostic and therapeutic standards. Additionally, school-based health education programs addressing skin diversity and chronic dermatological conditions seem to be a particularly relevant initiative given the recent public debate in Poland surrounding the introduction of health education as a school subject. Projects such as the AAD’s “Good Skin Knowledge” curriculum could help reduce stigmatization and increase awareness of AA among children and adolescents.

This study is subject to several limitations, one of which is the potential for selection bias. We acknowledge that recruiting participants from both a patient advocacy organization and a specialist dermatology center may have introduced selection bias as these individuals likely represent patients with greater disease severity, higher health literacy, and stronger engagement in disease management compared to the general AA population. Although no direct comparisons of recruitment sources in AA have been made to date, studies regarding other conditions demonstrate that patient advocacy organization members tend to have more extensive disease, higher income, and greater treatment awareness than non-members identified from the general population, while survey respondents recruited through advocacy groups report significantly worse quality-of-life scores and higher symptom burden compared to tertiary center patients [28,29]. Notably, a large UK population-based study found that people of higher social deprivation were less likely to be referred for specialist dermatology review, suggesting that clinic-based recruitment may systematically underrepresent socioeconomically disadvantaged patients [30]. In addition, a large European population-based study found that 44.7% of individuals with alopecia did not consult any healthcare professional, suggesting that a substantial proportion of patients with milder disease are unlikely to be captured through clinic- or advocacy-based recruitment [31]. Consequently, our findings may overestimate the quality-of-life burden experienced by the broader alopecia areata population. Thus, future studies employing population-based sampling strategies are warranted to improve generalizability.

## 5. Conclusions

Our findings highlight the multidimensional burden of alopecia areata in Poland and the challenges in delivering optimal care. While diagnostic and therapeutic practices are broadly aligned with international consensus, important gaps persist in the use of severity assessment tools, the integration of psychosocial evaluation, and access to advanced therapies. The recent introduction of ritlecitinib marks a long-awaited milestone, finally placing Poland on par with global standards of care and offering hope to patients with severe disease. However, improving outcomes will require more than pharmacological advances. A comprehensive, interdisciplinary approach—combining evidence-based medical treatment with psychological support, educational initiatives, and patient advocacy—is essential to address the full spectrum of needs faced by individuals with alopecia areata. Only by uniting these elements can we begin to dismantle the stigma, alleviate the psychosocial burden, and provide truly holistic care for this vulnerable patient population.

## Data Availability

The datasets generated during and/or analyzed during the current study are available from the corresponding author on reasonable request. The data are fully anonymized to ensure patient privacy.

## Supplementary Materials

The following supporting information can be downloaded at https://www.mdpi.com/article/10.3390/jcm15155993/s1. Supplement S1. Item-by-item SF-36 questionnaire results.

## Abbreviations

The following abbreviations are used in this manuscript:

AA	Alopecia Areata
SALT	Severity of Alopecia Tool
AAS	Alopecia Areata Scale
EMA	European Medicines Agency
DLQI	Dermatology Life Quality Index
CDLQI	Children’s Dermatology Life Quality Index
AAPPO	Alopecia Areata Patient Priority Outcomes
WPAI+CIQ	Work Productivity and Activity Impairment Questionnaire and Classroom Impairment Questions
SF-36	36-Item Short Form Survey
JAK	Janus Kinase
ASIR	age-standardized incidence rate
FDA	U.S. Food and Drug Administration
NAAF	National Alopecia Areata Foundation

## Appendix A

**Table A1:** Concordance and deviations between official guidelines and real-world treatment patterns in alopecia areata management among Polish physicians.

Clinical Scenario	Guideline Recommendation	Most Frequent Treatments in Our Study	Concordance/Deviation
**Limited AA** **(SALT ≤ 20, scalp)**	High-potency topical corticosteroids as first-line; intralesional triamcinolone in ≥13 yrs; contact immunotherapy optional, not first-line; calcineurin inhibitors discouraged	≥12 yrs: topical steroids 61.22% (*n* = 60); <12 yrs: topical steroids 62.89% (*n* = 61); second-line: corticosteroid + contact sensitizer (16.33%, 13.40%)	**High concordance** (topical steroids first-line). Earlier than recommended use of contact immunotherapy as second-line.
**Beard AA**	First-line: topical corticosteroids; calcineurin inhibitors not recommended	Topical steroids 57.14% (*n* = 56); calcineurin inhibitors 17.35% (*n* = 17); combination 12.24% (*n* = 12)	**Concordant for steroids**; **deviation:** relatively frequent use of calcineurin inhibitors despite lack of recommendation
**Eyebrow AA**	First-line: topical corticosteroids; prostaglandin analogues optional for eyelashes; calcineurin inhibitors discouraged	≥12 yrs: steroids 51.02% (*n* = 50), calcineurin inhibitors 25.51% (*n* = 25); <12 yrs: steroids 45.45% (*n* = 45), calcineurin inhibitors 35.35% (*n* = 35)	**Partial concordance** (steroids preferred), but **notable deviation:** substantial use of calcineurin inhibitors
**Severe localized AA** **(≥12 yrs)**	Systemic therapy required if SALT ≥20; systemic glucocorticosteroids, cyclosporine, methotrexate; JAK inhibitors preferred where available	Systemic GKS 29.47% (*n* = 28); topical steroids 18.85% (*n* = 18); cyclosporine 13.68% (*n* = 13)	**Concordant:** systemic therapy prioritized **Deviation:** topical steroids still used in severe cases; 2 JAK inhibitors reported
**Severe localized AA (<12 yrs)**	Systemic therapy considered; cautious use due to age; topical steroids may be continued in limited cases	Topical steroids 34.38% (*n* = 33); systemic GKS 18.75% (*n* = 18); cyclosporine + GKS 11.46% (*n* = 11)	**Mixed concordance:** systemic therapy used, but topical steroids remain dominant.
**Severe generalized AA (≥12 yrs)**	First-line systemic therapy; JAK inhibitors preferred; systemic GKS and cyclosporine as alternatives	Systemic GKS 45.16% (*n* = 42); cyclosporine 19.35% (*n* = 18); topical steroids 7.53% (*n* = 7)	**Concordant**: prioritizing systemic therapy **Deviation**: 2 JAK inhibitors reported
**Severe generalized AA (<12 yrs)**	Systemic therapy; topical steroids insufficient	Systemic GKS 33.68% (*n* = 32); cyclosporine 16.84% (*n* = 16); cyclosporine + GKS 12.63% (*n* = 12); topical steroids 12.63% (*n* = 12)	**Concordant:** emphasis on systemic therapy; some persistence of topical therapy despite severity

## References

[1] Zhou C., Li X., Wang C., Zhang J. (2021). Alopecia Areata: An Update on Etiopathogenesis, Diagnosis, and Management. Clin. Rev. Allergy Immunol..

[2] Pratt C.H., King B.A., Messenger A.G., Christiano A.M., Sundberg J.P. (2017). Alopecia Areata. Nat. Rev. Dis. Primers.

[3] Strazzulla L.C., Wang E.H.C., Avila L., Lo Sicco K., Brinster N., Christiano A.M., Shapiro J. (2018). Alopecia areata: Disease characteristics, clinical evaluation, and new perspectives on pathogenesis. J. Am. Acad. Dermatol..

[4] Minokawa Y., Sawada Y., Nakamura M. (2022). Lifestyle Factors Involved in the Pathogenesis of Alopecia Areata. Int. J. Mol. Sci..

[5] Simakou T., Butcher J.P., Reid S., Henriquez F.L. (2019). Alopecia areata: A multifactorial autoimmune condition. J. Autoimmun..

[6] Rajabi F., Drake L.A., Senna M.M., Rezaei N. (2018). Alopecia areata: A review of disease pathogenesis. Br. J. Dermatol..

[7] Gilhar A., Laufer-Britva R., Keren A., Paus R. (2019). Frontiers in alopecia areata pathobiology research. J. Allergy Clin. Immunol..

[8] Torales J., Castaldelli-Maia J.M., Ventriglio A., Almirón-Santacruz J., Barrios I., O’Higgins M., García O., Navarro R., Melgarejo O., Jafferany M. (2022). Alopecia areata: A psychodermatological perspective. J. Cosmet. Dermatol..

[9] Villasante Fricke A.C., Miteva M. (2015). Epidemiology and burden of alopecia areata: A systematic review. Clin. Cosmet. Investig. Dermatol..

[10] Meah N., Wall D., York K., Bhoyrul B., Bokhari L., Sigall D.A., Bergfeld W.F., Betz R.C., Blume-Peytavi U., Callender V. (2020). The Alopecia Areata Consensus of Experts (ACE) study: Results of an international expert opinion on treatments for alopecia areata. J. Am. Acad. Dermatol..

[11] Rakowska A., Rudnicka L., Olszewska M., Bergler-Czop B., Czuwara J., Brzezińska-Wcisło L., Narbutt J., Placek W., Zegarska B. (2023). Alopecia areata. Diagnostic and therapeutic recommendations of the Polish Dermatological Society. Part 1: Treatment. Dermatol. Rev..

[12] Muntyanu A., Gabrielli S., Donovan J., Gooderham M., Guenther L., Hanna S., Lynde C., Prajapati V.H., Wiseman M., Netchiporouk E. (2023). The burden of alopecia areata: A scoping review focusing on quality of life, mental health and work productivity. J. Eur. Acad. Dermatol. Venereol..

[13] Lacarrubba F., Dall’Oglio F., Rita Nasca M., Micali G. (2004). Videodermatoscopy enhances diagnostic capability in some forms of hair loss. Am. J. Clin. Dermatol..

[14] Rudnicka L., Olszewska M., Rakowska A. (2012). Atlas of Trichoscopy: Dermoscopy in Hair and Scalp Disease.

[15] Inamadar A.C., Palit A., Shivanna R., Deshmukh N.S. (2011). Light microscopy of the hair: A simple tool to “untangle” hair disorders. Int. J. Trichology.

[16] Rudnicka L., Rakowska A., Kurzeja M., Olszewska M. (2013). Hair shafts in trichoscopy: Clues for diagnosis of hair and scalp diseases. Dermatol. Clin..

[17] Guttikonda A.S., Aruna C., Ramamurthy D.V., Sridevi K., Alagappan S.K. (2016). Evaluation of Clinical Significance of Dermoscopy in Alopecia Areata. Indian J. Dermatol..

[18] Sibbald C. (2023). Alopecia Areata: An Updated Review for 2023. J. Cutan. Med. Surg..

[19] King B.A., Mesinkovska N.A., Craiglow B., Kindred C., Ko J., McMichael A., Shapiro J., Goh C., Mirmirani P., Tosti A. (2022). Development of the alopecia areata scale for clinical use: Results of an academic-industry collaborative effort. J. Am. Acad. Dermatol..

[20] Rakowska A., Rudnicka L., Olszewska M., Bergler-Czop B., Czuwara J., Brzezińska-Wcisło L., Narbutt J., Placek W., Zegarska B. (2023). Alopecia areata. Diagnostic and therapeutic recommendations of the Polish Society of Dermatology. Part 2: Treatment. Dermatol. Rev..

[21] Ministry of Health of Poland Notice of the Minister of Health of 17 June 2025, on the List of Reimbursed Medicinal Products, Foods for Special Medical Purposes, and Medical Devices Effective as of 1 July 2025. https://dziennikmz.mz.gov.pl/legalact/2025/39/.

[22] Darwin E., Hirt P.A., Fertig R., Doliner B., Delcanto G., Jimenez J.J. (2018). Alopecia Areata: Review of Epidemiology, Clinical Features, Pathogenesis, and New Treatment Options. Int. J. Trichology.

[23] Zhou J., Liang L., Zhang H., Liu M., Zhu Z., Leng L., Li J. (2025). Global Burden of Alopecia Areata and Associated Diseases: A Trend Analysis From 1990 to 2021. J. Cosmet. Dermatol..

[24] Vahdat V., Griffin J.A., Stahl J.E., Yang F.C. (2018). Analysis of the effects of EHR implementation on timeliness of care in a dermatology clinic: A simulation study. J. Am. Med. Inform. Assoc..

[25] European Medicines Agency Olumiant (Baricitinib). EMA/480402/2023. https://www.ema.europa.eu/en/medicines/human/EPAR/olumiant.

[26] O’Connell K.A., Ramos V., Giefer J., Martinez A., van den Bogert K., Boiko S. (2024). The American Academy of Dermatology’s “Good Skin Knowledge” program enhances children and adolescents’ confidence regarding dermatologic knowledge. Pediatr. Dermatol..

[27] Ellison A., Kranz D., Mesinkovska N.A., Christiano A.M., Shapiro J., Norris D.A. (2020). Forging the Future: 2018 Alopecia Areata Research Summit Summary Report. J. Investig. Dermatol. Symp. Proc..

[28] Yu J., Paranagama D., Parasuraman S. (2020). Recruitment strategies and geographic representativeness for patient survey studies in rare diseases: Experience from the living with myeloproliferative neoplasms patient survey. PLoS ONE.

[29] Prummer C.M., Kerezoudis P., Tombers N.M., Peris-Celda M., Link M.J., Carlson M.L. (2019). Influence of Selection Bias in Survey Studies Derived From a Patient-Focused Organization: A Comparison of Response Data From a Single Tertiary Care Center and the Acoustic Neuroma Association. Otol. Neurotol..

[30] Harries M., Macbeth A.E., Holmes S., Chiu W.S., Gallardo W.R., Nijher M., de Lusignan S., Tziotzios C., Messenger A.G. (2022). The epidemiology of alopecia areata: A population-based cohort study in UK primary care. Br. J. Dermatol..

[31] Adams C., Nassar E.-L., Rice D.B., Wurz A., Thombs B.D., Cook V., Gietzen A., Gottesman K., Guillot G., Lawrie-Jones A. (2026). Strengthening rheumatology research through meaningful engagement of people with lived experience. Lancet Rheumatol..

