# Supplementary material for: Assessing Alopecia Areata Management in Poland: Challenges in Medical Practices and Patient Care
Source: J Clin Med. 2026 Aug 1;15(15):5993. doi: 10.3390/jcm15155993 (PMC13466620; doi:10.3390/jcm15155993)
Supplement: Supplementary file 1 [file jcm-15-05993-s001.zip › jcm-4420721-supplementary.pdf]

Supplement S1. Item-by-item SF-36 questionnaire results.

Item	Response	%
In general, I would say that my health is:	Excellent	2,67
	Very good	18,72
	Good	40,11
	Not very good	31,02
	Bad	7,49
Compared to the period a year ago, I would rate my current health:	Much better now than a year ago	5,38
	Currently slightly better than last year	11,29
	Almost the same as last year	47,31
	Currently slightly worse than last year	20,43
	Currently significantly worse than last year	15,59

Does your health condition currently limit you in performing these activities? If so, to what extent?	Yes, very restrictive (%)	Yes, slightly limits (%)	No, it does not limit(%)
Activities that require intense effort, e.g. running, lifting heavy objects, strenuous sports activities	29,95	36,36	33,69
Moderate activities, e.g. moving the table, vacuuming, swimming, light gardening	8,56	28,34	63,10
Lifting or carrying shopping bags	6,45	20,43	73,12
Climbing several flights of stairs	8,11	29,73	62,16
Climbing one flight of stairs	4,28	11,76	83,96
Bending down or kneeling	11,29	24,19	64,52
Walking more than 1 kilometer	8,06	20,97	70,97
Walking several hundred meters	5,91	13,44	80,65
Walking a distance of 100 meters	4,84	10,75	84,41
Washing and dressing	7,57	12,97	79,46

Have you experienced any of the following problems at work or in your daily activities in the last 4 weeks due to your physical health problems?	All the time	Most of the time	Sometimes	Seldom	Never
Reduce the amount of time you spend working or doing other activities	7,07	14,13	27,17	19,57	32,07
Achieving less than you would like	11,35	18,38	33,51	19,46	17,30
Limiting the type of work or other activities performed	8,15	16,30	32,61	18,48	24,46
You had difficulty doing work or other activities (e.g., it required increased effort)	8,60	16,67	33,33	17,74	23,66

Have you experienced any of the following problems in your work or daily activities in the last 4 weeks due to emotional problems (e.g. depression, anxiety)?	All the time	Most of the time	Sometimes	Seldom	Never
Limit the amount of time spent on work or other activities	13,51	19,46	28,65	20,54	17,84
Achieving less than you would like	21,08	24,32	27,57	12,97	14,05
You have performed work or other activities less diligently than usual	15,14	21,08	33,51	13,51	16,76

Item	Answers	%
Over the past 4 weeks, to what extent has your physical health or emotional problems affected your normal social activities in your circle of family, friends, neighbors, or other groups?	Not at all	10,70
	Slightly	20,86
	Moderately	20,32
	To a large extend	32,09
	Very much	16,04
How much physical pain have you felt in the last 4 weeks?	Any	21,51
	Very mild	19,35
	Mild	18,82
	Moderate	29,03
	Strong	9,68
	Very strong	1,62
How much pain has interfered with your normal work (including working outside and at home) in the last 4 weeks?	Not at all	32,09
	Somewhat	24,60
	Moderately	27,81
	To a large extend	11,76
	Very much	3,74

The following questions are about how you have felt and how you have been doing in the last 4 weeks.	All the time	Most of the time	Sometimes	Seldo m	Never
You felt full of life	5,88	19,79	34,22	30,48	9,63
You were very nervous	14,59	38,38	35,14	9,19	2,70
You felt so depressed that nothing could cheer you up	10,75	19,89	38,71	16,67	13,98
You felt calm and gentle	2,69	19,89	38,71	28,49	10,22
You had a lot of energy	4,86	13,51	33,51	32,43	15,68
You felt discouraged and depressed	13,98	31,18	32,80	13,98	8,06
You felt exhausted	18,92	28,11	35,68	11,89	5,41
You were happy	5,41	20,00	40,54	22,16	11,89
You felt tired	20,86	33,69	33,16	9,09	3,21

Item	Answers	%
For how long in the last 4 weeks have your physical health or emotional problems interfered with your social activities (such as visits from friends, relatives, etc.)?	All the time	8,02
	Most of the time	28,34
	Sometimes	37,97
	Seldom	15,51
	Never	10,16

How true or false is each of the following statements to you?	Definitely true	Mostly true	I do not know	Mostly false	Definitely false
I seem to get sick more easily than other people	24,32	28,11	20,00	18,92	8,65
I'm just as healthy as other people I know	6,56	22,95	27,32	24,04	19,13
I expect my health to deteriorate	11,96	26,63	27,72	13,59	20,11
My health is excellent	3,23	18,28	16,13	26,88	35,48
